# Phylogenetic analyses suggest multiple changes of substrate specificity within the Glycosyl hydrolase 20 family

**DOI:** 10.1186/1471-2148-8-214

**Published:** 2008-07-22

**Authors:** Jari Intra, Giulio Pavesi, David S Horner

**Affiliations:** 1 Dipartimento di Scienze Biomolecolari e Biotecnologie, Università di Milano, Via Celoria 26, 20133 Milano, Italy

## Abstract

**Background:**

Beta-N-acetylhexosaminidases belonging to the glycosyl hydrolase 20 (GH20) family are involved in the removal of terminal β-glycosidacally linked N-acetylhexosamine residues. These enzymes, widely distributed in microorganisms, animals and plants, are involved in many important physiological and pathological processes, such as cell structural integrity, energy storage, pathogen defence, viral penetration, cellular signalling, fertilization, development of carcinomas, inflammatory events and lysosomal storage diseases. Nevertheless, only limited analyses of phylogenetic relationships between GH20 genes have been performed until now.

**Results:**

Careful phylogenetic analyses of 233 inferred protein sequences from eukaryotes and prokaryotes reveal a complex history for the GH20 family. In bacteria, multiple gene duplications and lineage specific gene loss (and/or horizontal gene transfer) are required to explain the observed taxonomic distribution. The last common ancestor of extant eukaryotes is likely to have possessed at least one GH20 family member. At least one gene duplication before the divergence of animals, plants and fungi as well as other lineage specific duplication events have given rise to multiple paralogous subfamilies in eukaryotes. Phylogenetic analyses also suggest that a second, divergent subfamily of GH20 family genes present in animals derive from an independent prokaryotic source. Our data suggest multiple convergent changes of functional roles of GH20 family members in eukaryotes.

**Conclusion:**

This study represents the first detailed evolutionary analysis of the glycosyl hydrolase GH20 family. Mapping of data concerning physiological function of GH20 family members onto the phylogenetic tree reveals that apparently convergent and highly lineage specific changes in substrate specificity have occurred in multiple GH20 subfamilies.

## Background

Carbohydrates are involved in many biological functions including maintenance of cell structural integrity, energy storage, pathogen defence, viral penetration, cellular signalling and fertilization. Enzymes specifically responsible for carbohydrate hydrolysis have been classified in 111 families of glycosyl hydrolases (GH) on the basis of amino acid sequence similarity [1,2]. Hexosaminidases belong to families GH3, GH20 and GH84 [2]. Among these, family 20 is of particular interest, and includes β-N-acetylhexosaminidases (β-hexosaminidase) (EC 3.2.1.52), enzymes that hydrolyze non-reducing terminal β-1,4 linked N-acetylglucosamine (GlcNAc) or β-N-acetylgalactosamine (GalNAc) residues of oligosaccharides and their conjugates, bacterial chitobiases (EC 3.2.1.30) and lacto-N-biasidase (EC 3.2.1.140). Crystal structures are known for numerous β-N-acetylhexosaminidases including the bacterial enzymes from *Serratia marcescens *[3] and *Streptomyces plicatus *[4], and the α- and β-chains of human lysosomal enzymes. The catalytic domain is an α/βTIM-barrel with the active site at the centre of the barrel complex [5-7].

In mammals, there are two major β-N-acetylhexosaminidase isoforms, named HEXA and HEXB, which reside in lysosomes and participate in the degradation of glycoproteins, glycolipids and glycosaminoglycans. HEXA is a heterodimer of subunits α (encoded by the gene *HEXA*) and β (encoded by the gene *HEXB*), whereas HEXB is a homodimer of β subunits. The subunits arose via a gene duplication event and the primary sequences are approximately 60% identical [8,9]. In particular, mutations in human *HEXA *and *HEXB *genes cause Tay-Sachs and Sandhoff, fatal neurodegenerative diseases, respectively [10,11]. Recent data suggest that lysosomal exoglycosidases along with many other factors may participate in the progression of development of tumor cells [12]. The potential involvement of a β-N-acetylhexosaminidase in fertilization in hamster [13] and human [14] has been also hypothesized.

Beta-hexosaminidases are also widely distributed in Insects. Several studies have led to the identification, molecular cloning and purification of β-N-acetylhexosaminidases in Lepidoptera like *Manduca sexta *[15], *Spodoptera frugiperda *[16], *Bombyx mori *[17], *Trichoplusia ni *[18], Diptera like *Drosophila melanogaster *[19,20] and *Aedes aegypti *[21] and more recently in the coleopteran *Tribolium Castaneum *[22]. The β-hexosaminidase activity of insects is of particular interest because of the role this glycosidase plays in the alteration of the structures of *N*-glycans generated in the cells [16,22-24] and in the chitin degradation processes [16,22,24,25]. Chitin, found in the cuticle of the integument and peritrophic membrane of the midgut, is a linear polymer of β-1,4 linked N-acetylglucosamine. Chitin degradation is catalyzed by chitinolytic enzymes in two successive steps: chitinase (EC 3.2.1.14) hydrolizes chitin into oligosaccharides of GlcNAc, then β-N-acetylhexosaminidase further degrades the oligomers into monomers. Because of the important role of chitin, β-N-acetylhexosaminidase is considered to be a potential target for insect control agents such as biopesticides [26,27]. Recently, the presence in the plasma membrane of spermatozoa of *Drosophila melanogaster *of two β-N-acetylhexosaminidases potentially involved in sperm-egg interactions has been demonstrated [20,28].

In crustaceans, β-N-acetylhexosaminidase has been shown to be important in the degradation of chitin forming the exoskeleton. Beta-N-acetylhexosaminidases have been purified and characterized in different species, such as *Euphausia superba *[29] and *Scylla serrata *[30].

In the ascidian *Phallusia mammillata *a β-N-acetylhexosaminidase present in the plasma membrane of spermatozoa might have a role in the primary binding between gametes [31].

In addition, an important function of a β-N-acetylhexosaminidase has been postulated in the anaerobic parasitic protozoan *Entamoeba histolytica*, the causative agent of infectious amoebiosis. Two β-N-acetylhexosaminidases have been identified, cloned and characterized. These enzymes are most probably involved in the destruction of glycoconjugates of the extracellular matrix components to pass basement membranes [32].

Hexosaminidase genes are also distributed among fungi and bacteria and the enzyme family plays an important physiological role in the natural recycling of chitin, a structural component of cell wall [33,34]. The molecular cloning of β-N-acetylhexosaminidases has been reported for several bacteria, such as *Alteromonas *sp. Strain O-7 [35], *Alteromonas *sp. Strain 10S-24 [36], *Serratia marcescens *[3], *Vibrio harveyi *[37], *Vibrio vulnificus *[38]*Enterobacter *sp. Strain G1 [39], *Cellulomonoas fimi *[40] and several fungi, such as *Trichoderma harzianum *[41], *Trichoderma atroviride *[42], *Aspergillus oryzae *[43], *Aspergillus nidulans *[44] and *Candida albicans *[45]. Beta-N-acetylhexosaminidase is an emerging target for the design of fungicides. In fact, several chitinolytic bacteria and fungi have been shown to be powerful biological control agents protecting for example plants against pathogens [41,46-48].

Plant β-hexosaminidases have been detected in a variety of tissues including seeds and leaves [49,50]. In particular, high levels of β-N acetylhexosaminidase activity have been detected in germinating seeds [50] suggesting a role in the storage of glycoproteins [51]. A function in defence processes has also been proposed, since several of the purified β-N-acetylhexosaminidases could be chitin-degrading enzymes [27,50]. More recently β-hexosaminidases of *Arabidopsis thaliana *have been cloned and characterized at the molecular level [52].

Despite the increasing number of hexosaminidase homologs that have been identified in different species, only restricted analyses of phylogenetic relationships between glycosyl hydrolases of the GH20 family have been conducted [3,16,22,24,31,40,53]. Because of their important roles in several biological processes, we have undertaken a more comprehensive analysis of the evolutionary history of the GH20 family. In this study we have identified, analyzed and characterized β-N-acetylhexosaminidases from prokaryotes and eukaryotes. We show that while the GH20 family is widely distributed among eubacteria, the observed taxonomic distribution is best explained by a combination of gene duplications and horizontal gene transfer events. Likewise, the limited occurrence of the family in archaebacteria is probably the result of lateral transfer from eubacteria. We trace gene duplication events both at basal and lineage-specific levels within eukaryotes and demonstrate that while the most widely studied GH20 family members derive from a single ancestral eukaryotic gene, a second subfamily of more divergent sequences present in at least the majority of metazoans was likely acquired from an independent prokaryotic source. Finally, we describe the patterns of conservation of protein features in numerous β-N-acetylhexosaminidase subfamilies and relate these features to the current understanding of GH20 family function, an important exercise given apparent convergence of physiological function of non-orthologous GH20 enzymes in eukaryotes.

## Results and discussion

### Beta-hexosaminidase sequences

The β-hexosaminidase sequences in our study were recovered from numerous organisms, including prokaryotic and eukaryotic species. Extensive similarity searches of the CAZy, Pfam and Swiss-Prot databases resulted in the identification of around 300 complete prokaryotic or eukaryotic β-hexosaminidase/β-hexosaminidase-like gene sequences encoding products belonging to the GH20 family (Table 1 and see additional file 1, 2, 3). Partial prokaryotic β-hexosaminidase sequences or those derived from whole genome shotgun (WGS) sequences that could not be reliably reassembled have not been used in this work. Vertebrate and non-vertebrate sequences show pairwise amino acid identity ranging from 20 to 40%, whereas identity is between 45% and 90% within mammals and from 20% to 75% between the members of the two insect orders examined here (Lepidoptera and Diptera).

**Table 1:** Eukaryotic β-hexosaminidase proteins belonging to the Glycosyl hydrolase family 20 used in Figure 4.

Number	Name	Accession number	Organism	Taxonomy
1	Hex1_At	AAD30612.1	*Arabidopsis thaliana*	Eukaryota Viridiplantae
2	Hex2_At	AAM91092.1	*Arabidopsis thaliana*	
3	Hex3_At	BAE99290.1	*Arabidopsis thaliana*	
4	Hex1_Os	BAC83175.1	*Oryza sativa*	
5	Hex2_Os	AAU44085.1	*Oryza sativa*	
6	Hex3_Os	BAD87534.1	*Oryza sativa*	
7	Hex4_Os	BAF11315.1	*Oryza sativa*	
8	Hex5_Os	AAV32135.1	*Oryza sativa*	
9	Hex_Aa	*	*Aedes aegypti*	Eukaryota Insecta
10	Hex_Ag	* (HEXO1)	*Anopheles gambiae*	
11	Hex1_Am	XP_624793.1 (HEXO2)	*Apis melliphera*	
12	Hex2_Am	XP_001122538.1 (FDL)	*Apis melliphera*	
13	Hex1_Bm	AAC60521.1 (HEXO1)	*Bombyx mori*	
14	Hex2_Bm	AAT99455.1 (HEXO2)	*Bombyx mori*	
15	Hex_Bman	AAG48701.1	*Bombyx mandarina*	
16	Hex_Cf	AAX94571.1	*Choristoneura fumiferana*	
17	Hex1_Dm	AAF47881.1 (HEXO1)	*Drosophila melanogaster*	
18	Hex2_Dm	AAM48390.1 (HEXO2)	*Drosophila melanogaster*	
19	Hex3_Dm	AAM29423.1 (FDL)	*Drosophila melanogaster*	
20	Hex1_Dp	XP_001352600.1 (HEXO1)	*Drosophila pseudoobscura*	
21	Hex2_Dp	XP_001354979.1 (HEXO2)	*Drosophila pseudoobscura*	
22	Hex3_Dp	XP_001361860.1 (FDL)	*Drosophila pseudoobscura*	
23	Hex_Ms	AAQ97603.1	*Manduca sexta*	
24	Hex_Of	ABI81756.1	*Ostrinia furnacalis*	
25	Hex1_Sf	ABA27427.1	*Spodoptera frugiperda*	
26	Hex2_Sf	ABB76924.1	*Spodoptera frugiperda*	
27	Hex_Tni	AAL82580.1	*Trichoplusia ni*	
28	Hex_Tc	XP_975660.1	*Tribolium castaneum*	
29	Hex_Bt	ABG66991.1	*Bos Taurus*	Eukaryota Mammalia
30	Hex_Cfa	ENSCAFP00000026129	*Canis familiaris*	
31	Hex_Fc	AAB30707.2	*Felis catus*	
32	Hex1_Hs	AAB00965.1 (HEXA)	*Homo sapiens*	
33	Hex2_Hs	AAA52645.1 (HEXB)	*Homo sapiens*	
34	Hex1_Mf	BAE01310.1	*Macaca fascicularis*	
35	Hex2_Mf	BAE02244.1	*Macaca fascicularis*	
36	Hex1_Mm	ENSMMUP00000014005	*Macaca mulatta*	
37	Hex2_Mm	ENSMMUP00000026294	*Macaca mulatta*	
38	Hex1_Md	ENSMODP00000002170	*Monodelphis domestica*	
39	Hex2_Md	ENSMODP00000002282	*Monodelphis domestica*	
40	Hex1_Mmu	AAC53246.1	*Mus musculus*	
41	Hex2_Mmu	AAA18776.1	*Mus musculus*	
42	Hex_Oa	ENSOANP00000024872	*Ornithorhynchus anaticus*	
43	Hex1_Pt	ENSPTRP00000012396	*Pan troglodytes*	
44	Hex2_Pt	ENSPTRP00000029093	*Pan troglodytes*	
45	Hex_Pp	CAH90623.1	*Pongo pygmaeus*	
46	Hex1_Rn	AAH82097.1	*Rattus norvegicus*	
47	Hex2_Rn	AAH79376.1	*Rattus norvegicus*	
48	Hex_Ss	CAA63123.1	*Sus scrofa*	
49	Hex1_Dr	AAH93192.1	*Danio rerio*	Eukaryota Teleostei
50	Hex2_Dr	*	*Danio rerio*	
51	Hex_Fr	*	*Fugu rubripes*	
52	Hex_Tn	*	*Tetraodon nigroviridis*	
53	Hex_Xt	*	*Xenopus tropicalis*	Eukaryota Amphibia
54	Hex_Gg	CAG32597.1	*Gallus gallus*	Eukaryota Aves
55	Hex_Cbr	*	*Caenorhabditis briggsae*	Eukaryota Nematoda
56	Hex1_Ce	AAA91263.1	*Caenorhabditis elegans*	
57	Hex_Ani	EAA63815.1	*Aspergillus nidulans FGSC A4*	Eukaryota Fungi
58	Hex_Aor	BAC41255.1	*Aspergillus oryzae*	
59	Hex_Cal	AAA34346.2	*Candida albicans*	
60	Hex_Cpo	ABB18373.1	*Coccidioides posadasii C735*	
61	Hex_Cba	ABG77528.1	*Cordyceps bassiana*	
62	Hex_Cne	AAW44323.1	*Cryptococcus neoformans var. neoformans JEC21*	
63	Hex_Mgr	XP_365077.1	*Magnaporthe grisea 70-15*	
64	Hex_Man	AAY17951.1	*Metarhizium anisopliae*	
65	Hex_Neo	AAU29327.1	*Neotyphodium sp. FCB-2004*	
66	Hex_Tat	AAT70229.1	*Trichoderma atroviride*	
67	Hex1_Th	AAB47060.1	*Trichoderma harzianum*	
68	Hex2_Th	AAB47061.1	*Trichoderma harzianum*	
69	Hex_Sja	AAW26910.1	*Schistosoma japonicum*	Eukaryota Trematoda
70	Hex_Fch	ABB86961.1	*Fenneropenaeus chinensis*	Eukaryota Crustacea
71	Hex1_Ehy	CAE46968.1	*Entamoeba histolytica*	Eukaryota Entamoebidae
72	Hex2_Ehy	CAD10500.3	*Entamoeba histolytica*	
73	Hex1_Cin	*	*Ciona intestinalis*	Eukaryota Ascidiacea
74	Hex2_Cin	*	*Ciona intestinalis*	
75	Hex_Pma	CAD57204.1	*Phallusia mammilata*	
76	Hex_Ddi	AAA33230.1	*Dictyostelium discoideum*	Eukaryota Mycetozoa

Asterisks indicate the novel amino acid sequences predicted in this study. Underlined accession numbers indicate hypothetical and/or unknown proteins in databases before our analysis

### Protein features and conserved

We observed hydrophobic sequences predicted to be a signal peptide with potential cleavage sites in the N-terminal regions of all metazoan β-hexosaminidases (not shown), except Hex3_Dm, Hex3_Dp, Hex2_Ag, that have a putative signal-anchor domain [20,24]. Moreover, SOSUI and HMMTOP analyses identified one or two putative transmembrane helices in all sequences, but these predictions have a low degree of confidence. Although β-hexosaminidases are typically soluble lysosomal proteins, they have been also found in body fluids in mammals [54-58], in insects [20,59-61] and in plants [52]. The presence of β-hexosaminidases in an extracellular compartment is not surprising. It is in fact known that lysosomal hydrolases can be released through constitutive secretion or through lysosomal exocytosis [57,62,63]. Furthermore, β-N-acetylhexosaminidases are present as intrinsic proteins of the plasma membrane in insects [20,24], in ascidians [31], in plants [52], in vertebrates, including man [13,14,64] as well as in a variety of human somatic cells [65,66]. Thus, it could be hypothesized that the membrane-associated β-N-acetylhexosaminidase might be generated by a deficient cleavage of the signal peptide sequence, as demonstrated for other transmembrane proteins that are also present as soluble enzymes [20,67-70]. If the signal peptide were not cleaved, the enzyme would be a type II plasma membrane protein with an anchor sequence, as demonstrated in a few human sperm and fruit fly proteins [14,20,28,70].

**Figure 1:**
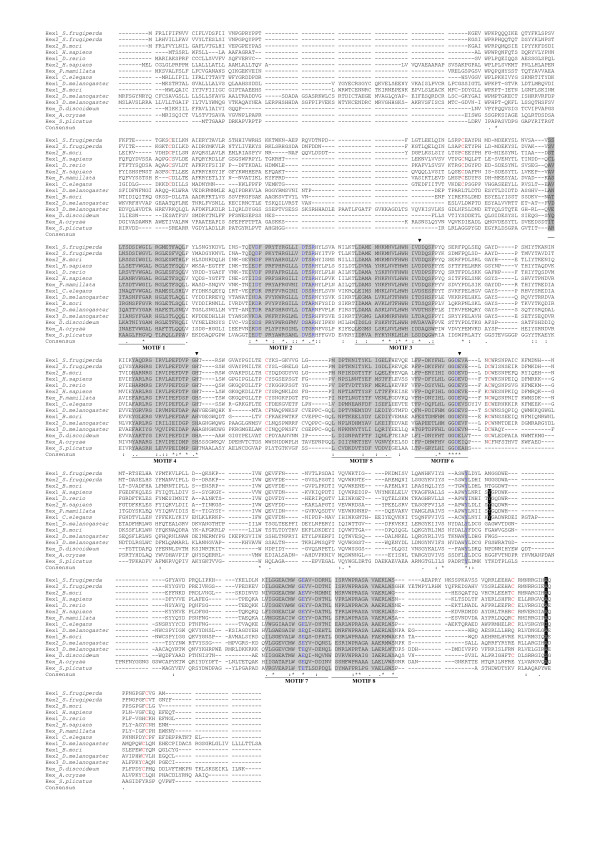
**Multiple sequence alignments of representative β-hexosaminidase proteins**. The sequences were aligned with Muscle [87]. The eight motifs known to be a signature for GH20 family are shaded in grey. Residues involved in substrate binding are blue and shaded in grey. The solid arrowheads indicated the amino acid residues that form the catalytic triad in human HEXB [5,6]. Tyrosine and alanine residues that play an important role in dimerization of two subunits in human HEXB [5] are white in black background. Cysteines involved in the formation of disulfide bonds in human HEXB [72] are red.

A multiple alignment of representative β-hexosaminidase protein sequences is shown in Figure 1. Amino acids belonging to six of the eight motifs known to be a signature for GH20 family are highly conserved (SPRINTS database; [71]), while amino acids in the first and fifth motifs are much less conserved. In particular we observe greater difference in β-hexosaminidase protein sequences of the dipteran species, while β-hexosaminidase of the lepidopteran *Spodoptera frugiperda *is very similar to mammalian β-hexosaminidases – as previously indicated [16]. Residues involved in substrate binding (Asp^196^, Asp^208^, Arg^211^, Asp^354^, Tyr^450^, Glu^491 ^– human HEXB numbering) and the triad aspartate (Asp^240^), histidine (His^294^), glutamate (Glu^355^) – known to be the amino acids involved in catalysis in human HEXB- are completely conserved [5,6]. Six cysteine residues (Cys^91^-Cys^137^, Cys^309^-Cys^360 ^and Cys^534^-Cys^551 ^– human HEXB numbering) form three disulfide bonds in human HEXB [72] and the third disulfide bond has been demonstrated to be important for enzymatic activity [73]. Only the cysteine residues involved in the second disulfide bond are fully conserved – suggesting that the disulfide bonds cannot be considered a conserved feature of all β-hexosaminidases. The tyrosine-456 and alanine-543 of β-chain in human HEXB play an important role in dimerization since they form hydrophobic interactions with the isoleucine-454, tyrosine-492 and threonine-496 of the second β-subunit [5]. As shown in Figure 1, tyrosine-456 is conserved in ascidians, nematodes and fish, while in fungi, insects and in the slime mould *Dictyostelium *it is replaced by different amino acid residues. Conversely, only in *Caenorhabditis *and *Dictyostelium *is the alanine residue not conserved. Although dimerization is considered a prerequisite for the formation of catalytically active enzymes in metazoans, our observations do not preclude that the enzymes dimerize and are active, as recently demonstrated in insects [16,20].

**Figure 2:**
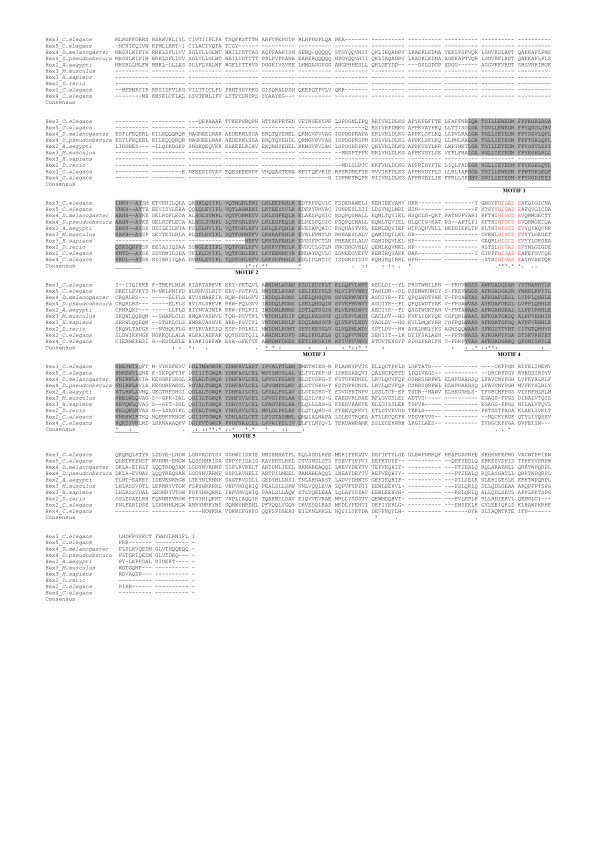
**Multiple sequence alignments of representative eukaryotic divergent GH20 sequence proteins**. Sequences were aligned with version Muscle [87]. Conserved motifs detected by MEME analysis are shaded in grey. Sequences containing the glutamate residue (E) known to be involved in the catalysis are red [5,6]. Hex2_Ce: *C. elegans *[GenBank: AAA96105.3]; Hex3_Ce: *C. elegans *[GenBank: CAI06053.1]; Hex4_Ce: *C. elegans *[GenBank: CAO72177.1]; Hex5_Ce: *C. elegans *[GenBank: CAA22078.2]; Hex4_Dm: *D. melanogaster *[GenBank: NP_650689.1]; Hex4_Dp: *D. pseudoobscura *[GenBank XP_001359965.1]; Hex2_Aa: *A. aegypti *[GenBank: XP_001649003.1]; Hex2_Dr: *D. rerio *[GenBank: NP_001070635.1]; Hex3_Mm: *M. musculus *[GenBank: BAE32455.1]; Hex3_Hs: *H. sapiens *[GenBank: BAB85072.1].

Figure 2 shows the multiple alignments of several eukaryotic β-hexosaminidases that have been defined as members of glycoside hydrolase family 20 in the CAZy and NCBI databases, but which do not show extensive global sequence similarity to other GH20 family members. DIALIGN found only a conserved region (seven amino acids) containing the glutamate residue that is involved in the catalytic mechanism of hexosaminidase [53]. We have recovered additional eukaryotic and prokaryotic β-hexosaminidase sequences (See additional file 2, 3) that appear to be homologs of these sequences. MEME, a tool to discover conserved motifs in a set of unaligned protein sequences (See Methods), detected the presence of conserved regions different from those known to be distinctive of β-hexosaminidases of GH20 (Figure 2). However, further research will be necessary to clarify the biochemical and functional role of these highly conserved regions.

Four gene products from *C. elegans *[GenBank: AAA96105.3, CAI06053.1, CAO72177.1, CAA22078.2] belong to this subfamily of GH20 and it has been recently demonstrated that they show β-hexosaminidase activity [53]. However, no data were presented on other amino acids involved in substrate binding and catalysis except the glutamate residue [5,6]. Further experiments are necessary to determine the enzyme activity of the probable orthologs to these *C. elegans *genes – which show most similarity to divergent GH20 proteins from low G+C gram positive bacteria, planctomycetes and a single crenarcheote (*Thermofilum pendens*) (See additional file 2, 3).

### Conservation and evolution of gene structure

**Figure 3:**
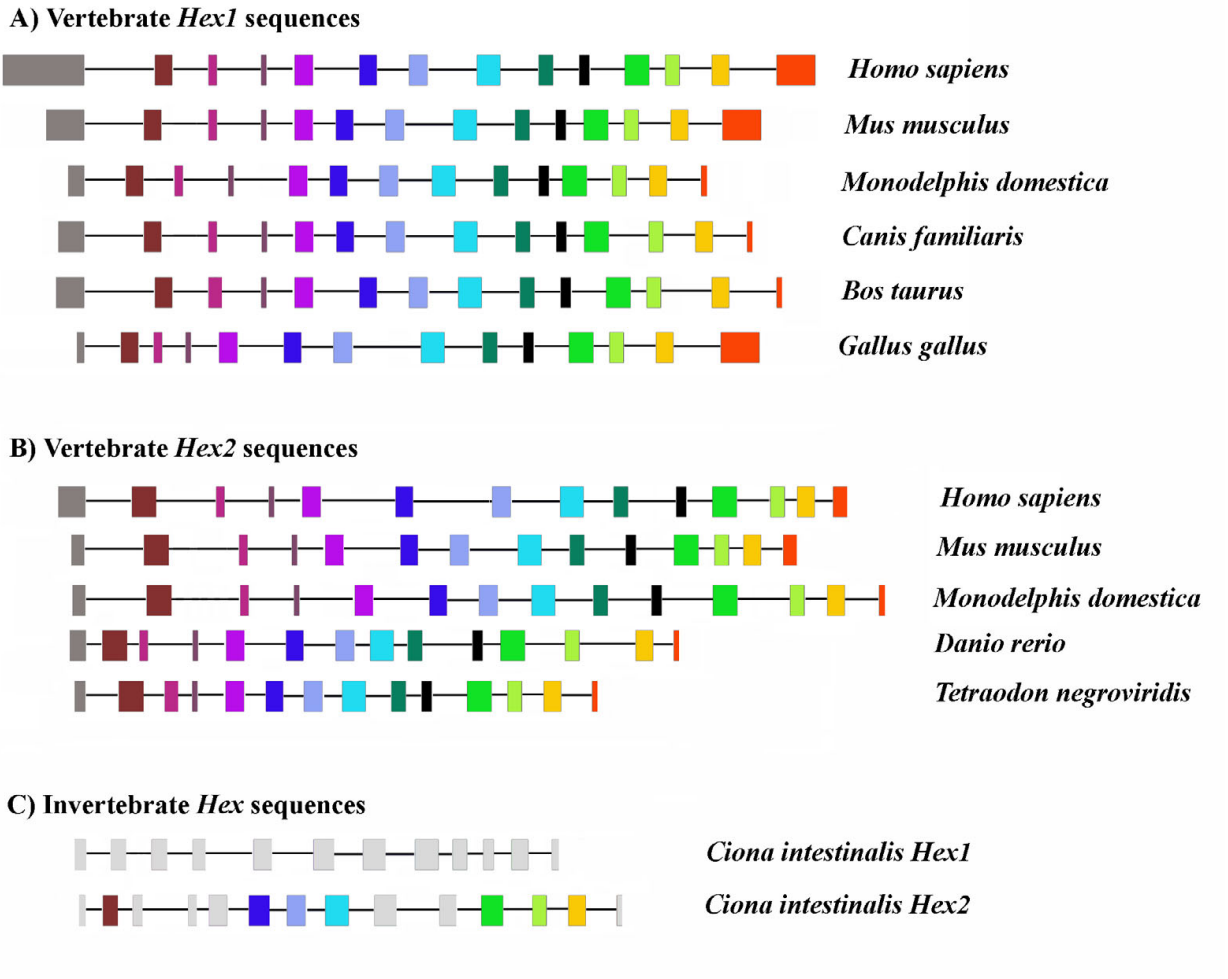
**Exon and intron patterns of representative genes of metazoan β-hexosaminidases of GH20 family**. Exon and intron patterns of representative genes of metazoan β-hexosaminidase of GH20 family, used in this study and drawn to scale relative to the number of nucleotides presenting each region (see additional file). Exons are represented with a rectangle, and homologous exons are depicted by a similar fill pattern. Gray filled rectangles represent non homologous exons in the ascidian *C. intestinalis*. Introns are depicted by a line between exons.

Figure 3 and Additional file 4 show the lengths of introns and exons of a representative sample of the β-hexosaminidase genes examined in this study, obtained with Exalign [74]. Within the vertebrate Hex1 and Hex2 subfamilies, the numbers and lengths of exons are remarkably conserved, together with intron phase. Vertebrate Hex1 and Hex2 isoforms are universally encoded by 14 exons. Among sampled species, only *Gallus gallus *shows a small degree of exon slippage of exons 4, 5 and 6 (which might be due to misannotations), while different lengths of initial and terminal exons (which include UTR sequences) are not unexpected. It is interesting that one of the β-hexosaminidase genes identified (Hex2_Ci) on the genome of the ascidian *C. intestinalis *has also conserved the size of several exons corresponding to vertebrate exons 2, 6, 7, 8,11, 12 and 13, consistent with this structure representing an ancestral chordate layout. Genes from more basal organisms (arthropods and nematodes) show a more variable structure in terms of intron number and position; no significant similarities with vertebrate genes were noted at the level of gene structure. Unsurprisingly, we observe similar gene structures also between pairs of Drosophila species, such as *D. melanogaster *and *D. pseudoobscura*, both in number or size of exons and in intron phase. On the other hand, in the nematode *C. elegans *only two β-hexosaminidases [GenBank: AAA91263.1, AAA96105.3] are encoded by 10 exons, while the other three β-hexosaminidases [GenBank: CAI06053.1, CAO72177.1, CAA22078.2] show a more variable structure in terms of exon number and position and intron phase (data not shown).

### Phylogenetic Analyses of β-hexosaminidase Sequences

**Figure 4:**
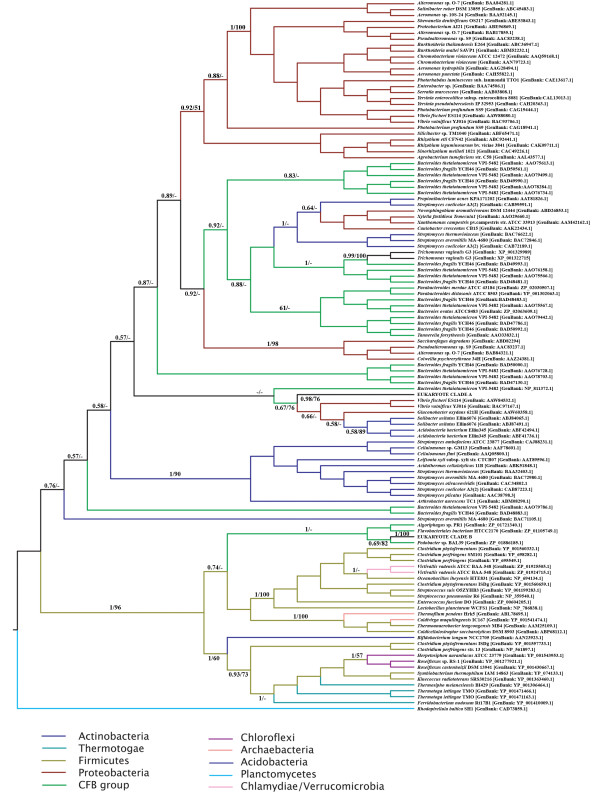
**Bayesian Consensus tree of representive Eukaryote and Prokaryote GH20 protein sequences**. The tree is rooted arbitrarily. Bayesian Posterior probabilities and Maximum-likelihood bootstrap proportions at important nodes are shown where above 0.5 or 50% respectively. All analyses were performed with the WAG amino acid substitution model and 1 invariable and 4 gamma distributed site rate categories.

Given the high degree of sequence divergence and indications of extensive saturation of substitutions at the nucleotide level (not shown), all phylogenetic analyses were performed on inferred full length amino acid sequences exhibiting overall co-linearity. Two separate datasets were prepared in order to maximize the number of unambiguously aligned amino acid residues used in phylogenetic analyses. The first set contained only eukaryote sequences showing overall co-linearity of alignment (76 sequences, 274 amino acid positions), the second included a the majority of eukaryote and prokaryote sequences – with only representatives of groups of highly similar sequences from closely related organisms removed to reduce the computational complexity of analysis of the large resulting dataset (233 sequences, 223 amino acid positions) – see materials and methods. A Bayesian phylogeny of the prokaryote and eukaryote dataset is shown in Figure 4, where the tree is rooted arbitrarily.

The GH20 family does not appear to be distributed evenly among bacterial phyla. Indeed, the majority of available sequences are derived from the Proteobacteria, Actinobacteria, Firimcutes, the Bacteroidetes/Chlorobi (CFB) group and Acidobacteria. Our phylogenetic analyses recover several well-supported clades containing members of well-defined taxonomic groups. For example, beta-proteobacterial and the majority of the gamma proteobacterial sequences are recovered as a well-supported monophyletic group which has a moderately supported association with some alpha-proteobacterial sequences. However, other gamma- and alpha-proteobacteria fall in disparate positions in the tree while Actinobacteria also fall in at least two clusters. Analogously, the Bayesian analysis recovers disparate clades of sequences derived from members of the Bacteroidetes/Chlorobi. These data suggest either ancient gene duplications or multiple horizontal gene transfer events within bacteria. We note that both Bayesian posterior probability and, in particular, bootstrap support for deep level relationships between groups is consistently low, prohibiting strong inferences regarding the evolution of the gene family in prokaryotes. Indeed analysis of constrained tree topologies using the Shimodaira/Hasegawa test suggested that a variety of relationships between the well defined groups indicated in Figure 3 were not significantly worse explanations of the data than the Bayesian consensus tree (not shown). It is thus impossible to judge whether an ancient origin of the GH20 family in bacteria was followed by multiple gene duplications and lineage specific losses of paralogs or whether the GH20 family arose within a discrete bacterial phylum and that the observed current distribution is a result of extensive lateral transfer coupled with occasional gene duplication. Interestingly, a well supported clade consisting mainly of sequences derived from Firmicutes (herein the "Firmicute" clade) also includes several sequences from CFB group bacteria, members of the Chlamydia/Verrucomicrobia group, a single Actinobacterial sequence, several members of the Thermatogae, several Chloroflexi and the few Archaebacterial sequences identified in this study. Taken together with the discontinuous presence GH20 family genes in Archaebacteria, Thermatogae, Chlamydiae and other bacterial taxa (and their apparent absence from other major bacterial groups (eg. Cyanobacteria), we suggest that extensive horizontal gene transfer has played a significant role in generating the observed taxonomic distribution of GH20 family members in prokaryotes.

**Figure 5:**
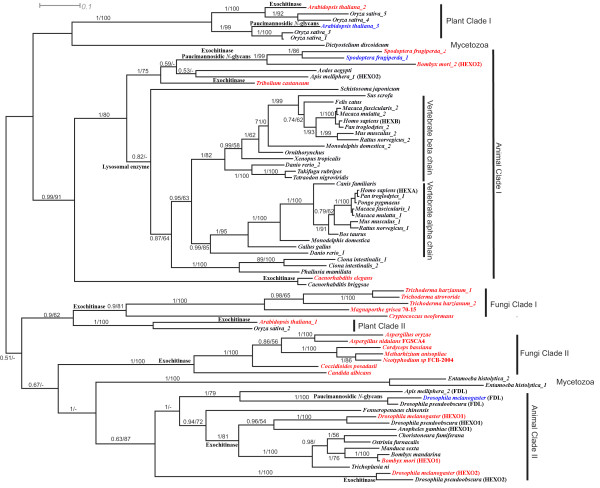
**Bayesian Consensus tree of Eukaryote GH20 protein sequences (Clade A)**. Analyses were performed on 274 unambiguously aligned amino acid positions from eukaryotic sequences passing tests of compositional homogeneity. The tree is rooted in a way to minimize inferred gene duplication events and maximize accordance with current understanding of organismal phylogenetic relationships. Bayesian Posterior probabilities and Maximum-likelihood bootstrap proportions are shown where above 0.5 or 50% respectively. All analyses were performed with the WAG amino acid substitution model and 1 invariable and 4 gamma distributed site rate categories. Proteins known to exhibit exochitinase activity are in red, whereas proteins involved in the formation of paucimannosidic *N*-glycans are in blue.

**Figure 6:**
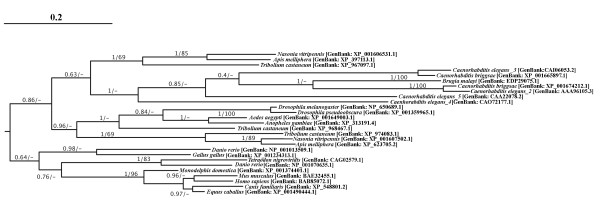
**Bayesian Consensus tree of Eukaryote GH20 protein sequences (Clade B)**. Experimental details as for Figure 4. Rooting is as inferred from the global analysis of GH20 sequences. Bayesian posterior probability and bootstrap support values are shown where over 0.5/50% respectively.

The eukaryotic sequences are recovered as two principal monophyletic groups, each with high bootstrap support and Bayesian posterior probability. The moderately-supported sister taxa to the first eukaryote clade (Eukaryote clade A (Figure 5) – which includes most of the sequences which have been subjected to functional analyses (see below)) is a paraphyletic assemblage of Acidobacterial and Proteobacterial species. While a second assemblage of divergent metazoan sequences (Eukaryote clade B – Figure 6) is recovered as a moderately supported sister to several CFB group proteins within the aforementioned "Firmicute" clade. Despite extensive constrained tree searches, we were unable to identify topologies portraying these two eukaryotic clades as a monophyletic group that were not excluded by the Shimodaira/Hasegawa test (not shown). While, the current data argue strongly for two acquisitions of GH20 family genes by eukaryotes (one in a common ancestor of plants, animals, fungi and mycetozoa, and another potentially in an early metazoan) the aforementioned considerations render speculation as to the nature of the prokaryote donors premature.

It is notable that sequences from the parasitic protist *Trichomonas vaginalis *are recovered within a well-supported clade of sequences from CFB group bacteria. Indeed, this case of probable prokaryote to eukaryote horizontal gene transfer was explicitly noted during the characterization of the genome of this parabasalid flagellate [75].

To better understand the evolution of the GH20 family within eukaryotes, we performed Bayesian – and maximum likelihood bootstrap – phylogenetic analyses of unambiguously aligned regions of inferred eukaryote clade A protein sequences (Figure 5). Vertebrate alpha and beta chains constitute monophyletic groups and are each others well supported sisters. The emergence of tunicate sequences as the monophyletic sister group of the vertebrate forms is consistent with a gene duplication in a common ancestor of vertebrates after the divergence of tunicates. Well-supported clusters of arthropod, nematode and schistosome sequences emerge basal to the tunicate clade.

A second, well-supported, cluster of arthropod sequences emerges in a distinct part of Eukaryote clade A (Figure 5). Indeed, the Bayesian analysis also recovers two clusters of plant, fungal and mycetozoan sequences – suggesting that a gene duplication event occurred in a common ancestor of plants, animals and fungi. However, it is not possible to position the root of the tree in such a way as to explain these multiple clusters with a single duplication event. We therefore investigated alternative hypotheses to explain the observed distribution of GH20 paralogs using constrained phylogenetic trees. We were unable to generate constrained topologies in which plant or arthropod sequences are monophyletic that were not statistically worse explanations of the data than the Bayesian topology according to the Shimodaira/Hasegawa (SH) test implemented in TREE-PUZZLE although all fungal sequences can be constrained as monophyletic as can mycetozoan (*Entamoeba *and *Dictyostelium*) sequences (which emerge close together in the bootstrap consensus tree). Several topologies describing plausible organismal relationships and invoking a single ancestral gene duplication in eukaryotes are not rejected by the SH test (See additional file 5) and the GH20 sequence data is thus compatible with current hypotheses of deep level organismal relationships within eukaryotes [76] and does not require invocation of eukaryote to eukaryote gene transfer as an explanation. While the monophyly, or otherwise, of fungal sequences cannot be established with confidence, the current data suggest that the observed diversity of arthropod and plant GH20 family genes result in part from ancient duplications that occurred before the divergence of plants and animals. However, subsequent "local" gene duplications have also occurred in both lineages. This phenomenon is particularly notable in plant clade I and in animal clade II – where successive gene duplications have given rise to *Hexo1*, *Hexo2 *and *fused lobes *(named *fdl*) genes.

Sequence similarity searches of the DOE Joint Genome Institute eukaryotic genome databases and other eukaryotic genome projects recover a large number of sequences that clearly derive from the GH20 family. Given the low quality of annotation and the widespread occurrence of potentially prokaryotic contaminants in such databases we have omitted such sequences from our phylogentic analyses. However, it is clear that the GH20 family is represented in all but a few major eukaryotic lineages. Taxa where complete or nearly complete genome sequences are available but where we were unable to recover traces of GH20 genes in at least one genome were restricted to divergent protists and unicellular organisms with secondarily reduced genomes: Diplomonads, Euglenozoa, Apicomplexa, and several Fungi (Saccharomyces, Schizosaccharomyces and Microsporidia).

### Two acquisitions of GH20 family genes by eukaryotes?

The Eukaryote clade B sequences (Figure 6) while conserved between each other, are characterized by generally high levels of divergence from the "Firmicute" clade in which they are embedded. Sequence similarity searches of the JGI eukaryotic genome databases revealed convincing traces of clade B sequences on the genomes of the cnidarian Nematostella [JGI:82909, JGI:245803], the placozoan Trichoplax [JGI:23741, JGI:2986, JGI:2419, JGI:51551], the mollusk Lottia [JGI:140764, JGI:235755] and the annelid Capitella [JGI:235755]. Unfortunately all associated gene predictions were of rather low quality and either incomplete or including apparently un-detected introns (or highly divergent insertions) and lacking in supporting EST evidence. Accordingly they were not used in phylogenetic analyses. However, the presence of highly conserved potentially coding sequences in these genomes (and the absence of such traces in the genome of the choanoflagellate Monosiga and other protist, fungal and plant genomes) is of some considerable interest. Our data are consistent with the proposition of Gutternigg et al. [53] that eukayotes have obtained GH20 family members from at least two phylogenetically distinct sources. The apparent absence of clade B sequences from non-metazoan genomes suggests a lateral transfer event into a basal metazoan, although an earlier acquisition – and subsequent loss from plant, protist and fungal genomes – can not be excluded. The strong statistical support for the monophyly of the "Firmicute" clade in which these and other more divergent sequences are embedded (Figure 4), our failure to identify acceptable topologies in which Eukaryote clade A and B sequences represent sister groups and the apparent absence of excessively long branches in this clade (not shown) lead us to prefer the hypothesis that these sequences represent a truly monophyletic assemblage rather than a phylogenetic artifact resulting from independent accelerations in evolutionary rates and resulting "long-branch attraction" between unrelated sequences.

### Evolution of substrate specificity in Eukaryote GH20 family members

The phylogenetic hypotheses presented here may be used to understand the evolution of GH20 family functionality in eukaryotes. While our phylogenetic reconstructions do not allow reliable rooting of the eukaryote tree or permit us to understand the evolutionary position of the more divergent GH20 isoforms, many clades and relationships are robustly supported. In this light it is informative to map functional data onto the trees. One of the most important differences between vertebrate and insects/worms/plants *N*-linked glycosylation is the presence of the paucimannosidic *N*-glycans structures in the second group. For example, the insect *fdl *genes are closely related to the *Hexo1 *and *Hexo2 *genes of insects. The *fdl *gene products are involved in the formation of paucimannosidic *N*-glycans [24,53], while *Hexo1 *and *Hexo2 *gene products (as well as fungal GH20 proteins [34]) are believed to function as degradative enzymes (exochitinases) [24]. Plant clade II sequences also have an exochitinase-like function, as recently demonstrated in *Arabidopsis thaliana *[52]. In this light it can be seen as likely that the ancestral activity for the plant, fungal and insect clade II sequences was likely to be as an exochitinase and that the *fdl *gene products have undergone a change in substrate specificity towards *N*-glycans. The animal Clade I sequences have been shown to be involved in the degradation of oligosaccharide chains of glycoproteins and glycolipids [7,77], although the proteins encoded by the lepidoptera *Spodoptera frugiperda *genes have been shown to be also responsible for the generation of paucimannosidic *N*-glycans [16,78], and might potentially represent a second example of functional shift within insects. Plant clade I sequences have been demonstrated to participate in the biosynthesis of truncated *N*-linked oligosaccharides and degradation of chitooligosaccharides [52,53]. Furthermore, the *C. elegans *hexosaminidase emerging in animal clade I likely has a role as a exochitinase [53] and may represent another example of a functional shift. We note that Drosophila species are not represented in clade I and speculate that their need for *N*-glycan-metabolizing β-hexosaminidases is fulfilled by the *fdl *gene product. Moreover, nematode and insect *N*-glycan hexosaminidases are membrane-bound proteins and are not localized in the lysosomes, while in plants the processing of *N*-glycans to paucimannosidic saccharides is in the vacuole [24]. An understanding of the functional roles of the Mycetozoan proteins which provide one with best candidates for the most ancient divergence in the eukaryote tree might be the key to understanding the nature of the ancestral enzyme activity. However, functional characterization of the slime mould *Dictyostelium *GH20 protein has not been published and biochemical data of the β-N-acetylglucosaminidases of *Entamoeba histolytica *do not allow strong inference as to the substrate specificity of these amoebozoan enzymes [79]. Furthermore, although we have observed high conservation of amino acids involved in substrate binding (see above), a crystal structure of a β-hexosaminidase involved in paucimannosidic *N*-glycans formation might provide precise information about the characteristics of active sites of β-hexosaminidases that display exochitinase activity. Despite the incompleteness of functional information, the phylogenetic tree presented in Figure 5 suggests that synthesis of paucimanosidic glycans is a derived characteristic that has evolved independently on at least 3 occasions. Finally, Gutternigg et al. [53] showed that two clade B *C. elegans *gene products [GenBank: AAA96105.3, CAI06053.1] are involved in the metabolism of paucimannosidic *N*-glycans, while other two nematode β-hexosaminidases have chitoligosaccharidase activity [GenBank: CAO72177.1; CAA22078.2]. The functional roles of the vertebrate sequences that appear to be most closely related to these divergent nematode genes (see above and additional file 3) remain obscure.

## Conclusion

Our phylogenetic analyses of GH20 family proteins provides new insights into evolutionary relationships and the history of the protein family and represent the first such detailed study of the GH20 protein family. We show that eukaryote sequences derive from two independent gene acquisitions. The most widely studied group of genes was present in a common ancestor of plants animals and fungi and the ancestral sequence underwent at least one gene duplication event early in eukaryote evolution gaving rise to at least two paralogs that evolved differential functions as degradative enzymes or a processing activity involved in the synthesis of paucimannosidic *N*-linked oligosaccharides. Despite these ancient roles, members of both subfamilies of eukaryote clade A have undergone apparent functional shifts, typified by the FDL proteins of insects which appear to be derived from exochitinase-like ancestors but which now play a role in the metabolism of paucimannisodic *N*-glycans. Conversely, the *C. elegans *sequence represented in Figure 5 is derived from paucimannisodic glycan proccessing enzymes, but functions as an exochitinase [53]. Indeed, the mammalian isoforms also derived from paucimannosidic *N*-glycans proccessing enzymes, but exhibit specificity towards the more complex oligosaccharides present in mammal cells. It remains unclear whether these differences reflect differential substrate specificities, or are more related to physiological patterns of expression.

Our data lend strong support to the contention of Gutternigg et al. [53] that more divergent GH20 family members from *C. elegans *and other metazoa are likely to be derived from a separate acquisition from prokaryotes. While the physiological roles of these proteins have not yet been studied experimentally outside of nematodes, it is unlikely that they should be involved in the production of paucimannosidic *N*-glycans in mammals, and it is thus probable that additional important roles for GH20 members remain to be discovered in animals.

## Methods

### Sequence retrieval

All characterized and complete β-N-acetylhexosaminidases protein sequences belonging to the GH20 family were retrieved from CAZy [2], Pfam [80] and Swiss-Prot [81] databases. In order to retrieve all the other β-N-acetylhexosaminidase sequences available we performed PSI-BLAST searches of the protein database throughout all organisms at NCBI [82] and through translated BLAST searches (tBLASTn) against the full genomic sequences available at the UCSC genome browser [83], using *Homo sapiens *protein sequences HEXA [GenBank: AAB00965.1] and HEXB [GenBank: AAA52645.1], *Arabidopsis thaliana *β-N-acetylhexosaminidase-like protein [GenBank: AAM61367.1], *Danio rerio *Zgc:112084 protein [GenBank: AAH93192.1], *Drosophila melanogaster *protein sequences HEXO1 [GenBank: AAF47881.1], HEXO2 [GenBank: AAM48390.1] and FDL [GenBank: AAM29423.1], and *Streptomyces plicatus *β-N-acetylhexosaminidase protein [GenBank: AAC38798.3] as queries, respectively. Only complete and representative protein sequences were employed for subsequent evolutionary study and protein analysis. For proteins with possible splice variants the longest predicted isoforms were used. A total of 223 protein sequences distributed across Eubacteria, archaebacteria and eukaryotes, were selected for the analysis of protein features and phylogenetic study (Table 1 and See additional file 1, 2, 3). In this study, we denote β-N-acetylhexosaminidase proteins as Hex followed by Arabic number when two or more β-N-acetylhexosaminidase proteins have been identified in the same organism and the abbreviation of genus and species name (e.g. *Bacteroides fragilis *NCTC 9343 β-N-acetylhexosaminidase will be denoted as Hex1_Bfr).

### Protein sequence analysis of β-N-acetylhexosaminidases

Beta-N-acetylhexosaminidase sequences were analyzed using SignalP 3.0 [84], with default options to predict signal peptide sequences; SOSUI [85] and HMMTOP [86] with default parameters for the prediction of transmembrane helices; SMART [87] to predict the domain architecture. Conserved motif analysis was performed by MEME program [88] using default settings.

### Multiple sequence alignment and phylogenetic analysis

In order to maximize the number of unambiguously aligned sites used for phylogenetic analysis of eukaryote sequences, two separate datasets were prepared. The first set contained only eukaryote sequences which passed tests of compositional homogeneity implemented in TREE-PUZZLE [89] while the second included available prokaryote sequences (several clusters of extremely closely related sequences from taxonomically similar bacteria were excluded to reduce the computational burden of phylogenetic analyses).

In both cases, inferred protein sequences were aligned with the software Muscle [90] and alignments refined manually. Unambiguously aligned regions were identified using the program GBlocks [91]. The resulting datasets contained 233 sequences with 223 amino acid positions (prokaryote and eukaryote dataset) and 76 sequences with 274 amino acid positions (eukaryote-only dataset). The WAG amino acid substitution model [92] with gamma distributed site rates and an invariable site category was used in all phylogenetic analyses as the Prottest software [93] indicated that this model provided the best fit to the data. Phylogenetic trees were estimated within the Bayesian statistical framework using a parallelized version of the program MrBayes v3.1 [94] on a cluster of processors running the linux operating system (2000000 generations with trees sampled every 50 generations). The first 5000 trees generated were excluded as "burnin" for the MCMC chains (likelihood stabilization was determined graphically). The program SEQBOOT from the PHYLIP [95] package was used to generate 100 pseudoreplicate datasets, maximum-likelihood trees were estimated for each replicate using the program PHYML [96] and consensus trees were inferred using the program CONSENSE. Constrained trees were generated manually and evaluated according to the Shimodaira/Hasegawa test of alternative tree topologies [97] as implemented in TREE-PUZZLE.

## Authors' contributions

JI performed database searches, conducted data analysis, sequence alignment and drafted the manuscript. GP participated in data analysis and manuscript revision. DSH conducted comparative genome analysis and evolution, sequence alignment, phylogenetic analysis and drafted the manuscript. All authors read and approved the final manuscript.

## Supplementary Material

Additional file 1 Eukaryotic GH20 protein sequences. All eukaryotic protein sequences of GH20 collected and used in this work. Click here for file

Additional file 2 Prokaryotic GH20 protein sequences. All prokaryotic protein sequences of GH20 collected and used in this work. Click here for file

Additional file 3 Divergent Eukaryotic GH20 protein sequences. Divergent eukaryotic protein sequences of GH20 collected and used in this work. Click here for file

Additional file 4 Gene structure of metazoan β-hexosaminidases. Lengths of exons and introns of representative metazoan β-hexosaminidases. Click here for file

Additional file 5 Alternative Hypotheses of relationships between major eukaryote GH20 clades. Schematic trees representing alternative hypotheses of relationships between major clades of eukaryotic GH20 family members. Hypotheses of relationships not excluded at the 5% confidence interval by the Shimodaira/Hasegawa test are depicted along with their log likelihoods, delta lnL and P-values. All analyses were performed with the WAG amino acid substitution model and 1 invariable and 4 gamma distributed site rate categories. Click here for file
