# Supplementary material for: Phylogenetic analyses suggest multiple changes of substrate specificity within the Glycosyl hydrolase 20 family
Source: BMC Evol Biol. 2008 Jul 22;8:214. doi: 10.1186/1471-2148-8-214 (PMC2492878; doi:10.1186/1471-2148-8-214)
Supplement: Additional file 4 — Gene structure of metazoan β-hexosaminidases. Lengths of exons and introns of representative metazoan β-hexosaminidases. [file 1471-2148-8-214-S4.doc]

| **Table 2** | | | | | | | | | | | | | | | | | | | | | | | | | | | |
| --- | --- | --- | --- | --- | --- | --- | --- | --- | --- | --- | --- | --- | --- | --- | --- | --- | --- | --- | --- | --- | --- | --- | --- | --- | --- | --- | --- |
| **Hexosaminidase** | **Exon**  **1** | **Intron**  **1** | **Exon**  **2** | **Intron**  **2** | **Exon**  **3** | **Intron**  **3** | **Exon**  **4** | **Intron**  **4** | **Exon**  **5** | **Intron**  **5** | **Exon**  **6** | **Intron**  **6** | **Exon**  **7** | **Intron**  **7** | **Exon**  **8** | **Intron**  **8** | **Exon**  **9** | **Intron**  **9** | **Exon**  **10** | **Intron**  **10** | **Exon**  **11** | **Intron**  **11** | **Exon**  **12** | **Intron**  **12** | **Exon**  **13** | **Intron**  **13** | **Exon**  **14** |
| **Vertebrate Hex1 sequences** | | | | | | | | | | | | | | | | | | | | | | | | | | | |
| Hex1_Hs | 754 | 19102 | 93 | 900 | 66 | 1821 | 47 | 512 | 111 | 1833 | 102 | 482 | 133 | 1258 | 181 | 944 | 87 | 289 | 73 | 975 | 184 | 201 | 91 | 684 | 105 | 1305 | 284 |
| Hex1_Mm | 310 | 14156 | 93 | 1113 | 66 | 1467 | 47 | 369 | 111 | 635 | 102 | 1188 | 133 | 1440 | 181 | 865 | 84 | 269 | 73 | 498 | 184 | 173 | 91 | 568 | 105 | 833 | 285 |
| Hex1_Md | 133 | 4530 | 96 | 895 | 66 | 882 | 47 | 1643 | 111 | 886 | 102 | 780 | 133 | 405 | 181 | 651 | 84 | 940 | 73 | 648 | 184 | 1557 | 91 | 259 | 105 | 1013 | 52 |
| Hex_Cfa | 253 | 15193 | 93 | 917 | 66 | 1686 | 47 | 502 | 111 | 890 | 102 | 465 | 133 | 616 | 187 | 985 | 87 | 265 | 73 | 711 | 184 | 213 | 91 | 424 | 105 | 1050 | 64 |
| Hex_Bt | 253 | 20519 | 93 | 1013 | 114 | 1581 | 47 | 507 | 111 | 866 | 102 | 822 | 133 | 570 | 181 | 904 | 87 | 248 | 73 | 650 | 184 | 143 | 91 | 918 | 105 | 1017 | 64 |
| Hex_Gg | 61 | 742 | 93 | 174 | 56 | 991 | 37 | 187 | 19 | 991 | 114 | 316 | 130 | 2610 | 181 | 1071 | 87 | 584 | 73 | 637 | 175 | 797 | 91 | 209 | 105 | 1467 | 184 |
| **Vertebrate Hex1 sequences** | | | | | | | | | | | | | | | | | | | | | | | | | | | |
| Hex2_Hs | 416 | 3768 | 146 | 4165 | 66 | 2988 | 47 | 256 | 111 | 8112 | 102 | 8185 | 130 | 1874 | 181 | 896 | 87 | 1617 | 73 | 433 | 175 | 1456 | 91 | 124 | 105 | 370 | 183 |
| Hex2_Mm | 245 | 3758 | 146 | 2843 | 66 | 1860 | 47 | 233 | 111 | 3292 | 102 | 1761 | 130 | 1663 | 181 | 664 | 87 | 1665 | 73 | 1289 | 175 | 733 | 91 | 154 | 105 | 207 | 183 |
| Hex2_Md | 230 | 4061 | 149 | 6637 | 66 | 1437 | 47 | 922 | 111 | 2294 | 102 | 1688 | 130 | 1805 | 181 | 1618 | 87 | 1678 | 73 | 2971 | 178 | 2454 | 91 | 256 | 105 | 810 | 40 |
| Hex2_Dr | 308 | 165 | 149 | 278 | 66 | 1326 | 47 | 123 | 111 | 1827 | 102 | 2685 | 133 | 100 | 181 | 126 | 87 | 2165 | 73 | 364 | 175 | 3040 | 91 | 1358 | 105 | 303 | 40 |
| Hex_Tn | 269 | 511 | 152 | 590 | 66 | 239 | 47 | 234 | 111 | 281 | 102 | 317 | 133 | 385 | 148 | 331 | 87 | 255 | 73 | 1026 | 175 | 481 | 91 | 276 | 105 | 247 | 64 |
| **Invertebrate Hex sequences** | | | | | | | | | | | | | | | | | | | | | | | | | | | |
| Hex1_Dm | 77 | 125 | 635 | 81 | 1175 |  |  |  |  |  |  |  |  |  |  |  |  |  |  |  |  |  |  |  |  |  |  |
| Hex2_Dm | 149 | 618 | 1538 | 71 | 182 |  |  |  |  |  |  |  |  |  |  |  |  |  |  |  |  |  |  |  |  |  |  |
| Hex3_Dm | 164 | 740 | 509 | 1309 | 730 | 1113 | 319 | 640 | 255 |  |  |  |  |  |  |  |  |  |  |  |  |  |  |  |  |  |  |
| Hex1_Dp | 80 | 156 | 635 | 60 | 1109 |  |  |  |  |  |  |  |  |  |  |  |  |  |  |  |  |  |  |  |  |  |  |
| Hex2_Dp | 176 | 586 | 1538 | 60 | 182 |  |  |  |  |  |  |  |  |  |  |  |  |  |  |  |  |  |  |  |  |  |  |
| Hex3_Dp | 164 | 723 | 494 | 1291 | 736 | 1123 | 319 | 637 | 255 |  |  |  |  |  |  |  |  |  |  |  |  |  |  |  |  |  |  |
| Hex1_Am | 147 | 135 | 123 | 407 | 447 | 99 | 513 | 114 | 93 |  |  |  |  |  |  |  |  |  |  |  |  |  |  |  |  |  |  |
| Hex2_Am | 252 | 639 | 225 | 694 | 117 | 104 | 372 | 140 | 291 | 283 | 261 | 189 | 173 |  |  |  |  |  |  |  |  |  |  |  |  |  |  |
| Hex3_Am | 615 | 2785 | 732 | 62 | 144 |  |  |  |  |  |  |  |  |  |  |  |  |  |  |  |  |  |  |  |  |  |  |
| Hex1_Ag | 78 | 365 | 1768 |  |  |  |  |  |  |  |  |  |  |  |  |  |  |  |  |  |  |  |  |  |  |  |  |
| Hex2_Ag | 234 | 779 | 687 | 70 | 159 | 63 | 1152 |  |  |  |  |  |  |  |  |  |  |  |  |  |  |  |  |  |  |  |  |
| Hex1_Ci | 138 | 449 | 112 | 421 | 165 | 457 | 150 | 921 | 159 | 440 | 174 | 369 | 93 | 261 | 115 | 236 | 63 |  |  |  |  |  |  |  |  |  |  |
| Hex2_Ci | 120 | 369 | 93 | 261 | 105 | 1658 | 87 | 136 | 111 | 273 | 114 | 429 | 112 | 752 | 165 | 315 | 150 | 511 | 158 | 476 | 174 | 398 | 93 | 507 | 105 | 200 | 57 |
|  |  |  |  |  |  |  |  |  |  |  |  |  |  |  |  |  |  |  |  |  |  |  |  |  |  |  |  |
| Exons and introns are shown in base pairs. | | | | | | | | | | | | | | | | | | | | | | | | | | | |
