# Supplementary figures and images for: Phylogenetic analyses suggest multiple changes of substrate specificity within the Glycosyl hydrolase 20 family
Source: BMC Evol Biol. 2008 Jul 22;8:214. doi: 10.1186/1471-2148-8-214 (PMC2492878; doi:10.1186/1471-2148-8-214)

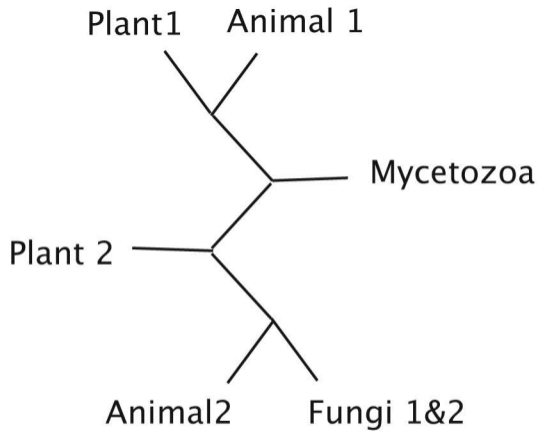

$\Delta \ln L = 18.08$   $pSH = 0.3400$

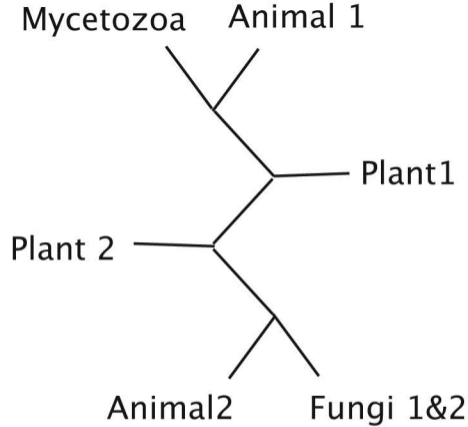

$\ln L = -19569.14$ ,  $pSH = 0.405$

Supplement: Additional file 5 — Alternative Hypotheses of relationships between major eukaryote GH20 clades. Schematic trees representing alternative hypotheses of relationships between major clades of eukaryotic GH20 family members. Hypotheses of relationships not excluded at the 5% confidence interval by the Shimodaira/Hasegawa test are depicted along with their log likelihoods, delta lnL and P-values. All analyses were performed with the WAG amino acid substitution model and 1 invariable and 4 gamma distributed site rate categories. [file 1471-2148-8-214-S5.pdf]
